# The Impact of the Covid-19 Pandemic and the Lockdown on the Health and Living Conditions of Undocumented Migrants and Migrants Undergoing Legal Status Regularization

**DOI:** 10.3389/fpubh.2020.596887

**Published:** 2020-12-16

**Authors:** Claudine Burton-Jeangros, Aline Duvoisin, Sarah Lachat, Liala Consoli, Julien Fakhoury, Yves Jackson

**Affiliations:** ^1^Institute of Sociological Research, University of Geneva, Geneva, Switzerland; ^2^Swiss NCCR “LIVES - Overcoming Vulnerability: Life Course Perspectives”, University of Geneva, Geneva, Switzerland; ^3^Center for the Interdisciplinary Study of Gerontology and Vulnerability, University of Geneva, Geneva, Switzerland; ^4^Faculty of Public Health and Policy, London School of Tropical Medicine and Hygiene, London, United Kingdom; ^5^Division of Primary Care Medicine, Geneva University Hospital and University of Geneva, Geneva, Switzerland

**Keywords:** COVID-19, migrant, impact, lockdown, undocumented, health, living conditions

## Abstract

**Introduction:** Undocumented migrants are at high risk of adverse consequences during crises because of a lack of access to essential securities and sources of support. This study aims to describe the impact of the COVID-19 crisis on the health and living circumstances of precarious migrants in Switzerland and to assess whether those undergoing legal status regularization fared better than undocumented migrants.

**Materials and methods:** This cross-sectional mixed methods study was conducted during the COVID-19 lockdown in April–May 2020. Undocumented and recently regularized migrants taking part in an ongoing cohort study were asked to respond to an online questionnaire. A subsample was selected to undergo semi-directed phone interviews.

**Results:** Overall, 117 of the 379 (30.9%) cohort study participants responded to the questionnaire. Seventeen interviews were conducted. Migrants faced cumulative and rapidly progressive difficulties in essential life domains. As a consequence, they showed high prevalence of exposure to COVID-19, poor mental health along with frequent avoidance of health care. Moreover, the loss of working hours and the related income overlapped with frequent food and housing insecurity. Around one participant in four had experienced hunger. Despite these unmet needs, half of the participants had not sought external assistance for reasons that differ by legal status. Both groups felt that seeking assistance might represent a threat for the renewal or a future application for a residency permit. While documented migrants were less severely affected in some domains by having accumulated more reserves previously, they also frequently renounced to sources of support.

**Conclusions:** The cumulated difficulties faced by migrants in this period of crisis and their limited search for assistance highlight the need to implement trust-building strategies to bridge the access gap to sources of support along with policies protecting them against the rapid loss of income, the risk of losing their residency permit and the exposure to multi-fold insecurities.

## Introduction

Since they affect everyone, catastrophes and crises are often thought of as levelers of inequalities in society. However, even though anyone can suffer consequences, the differentiated distribution of resources across social groups make some better able to cope with these consequences than others. The COVID-19 crisis has rapidly confirmed that infectious disease outbreaks reinforce social inequalities (1). After an initial focus on the medical crisis induced by the spread of the new virus, the social and economic impact of the pandemic quickly became apparent among the most vulnerable groups of the population, notably undocumented migrants (2). The pandemic, and its associated public health measures, have thus acted as a magnifying lens on pre-existing structural risks and vulnerability (1, 3).

Academic researchers and advocates well aware of undocumented migrants' precarious living conditions rapidly emphasized their unique position amidst the pandemic management. The World Health Organization and scholars have advocated for increased efforts to respond to the specific needs of these groups (2, 4, 5). Indeed undocumented migrants' pre-existing working and housing conditions increase their risk to be infected by the virus, while along recurrently observed social reactions toward infectious diseases (6) they are at additional risk to be blamed for their role in the dissemination of the virus (2). Policy recommendations to protect undocumented migrants were thus formulated in different contexts, as for example by the Migrant and Ethnic Health section of the European Public Health Association and the Platform for International Cooperation on Undocumented Migrants (PICUM), including requests for improved access to health care (2).

To this day, several editorials or perspectives pieces in the medical literature (2, 7, 8) have called for attention toward undocumented migrants in the COVID-19 context. However, little empirical evidence is reported, as by definition this population is difficult to reach and knowledge on their circumstances is limited even in regular circumstances. Taking advantage of an on-going prospective study on undocumented migrants in Geneva Switzerland (9), we have been able to collect quantitative and qualitative data among over 100 persons in the midst of the local lockdown measures. The longitudinal project aims at assessing how a regularization policy is impacting the health and socioeconomic conditions of those who obtained a legal status, as compared to a control group of undocumented migrants. The objective of this paper is to describe the impact of the COVID-19 crisis on the health and living circumstances of precarious migrants in Geneva, Switzerland and to assess whether those undergoing legal status regularization fared better than undocumented migrants.

## The COVID-19 Vulnerability of Undocumented Migrants

Vulnerable groups of population including migrants, have experienced an excess in COVID-19 morbidity and mortality (10, 11). While increased morbidity and mortality secondary to the SARS-CoV-2 infection have primarily affected people with pre-existing chronic diseases and older age, delayed non-medical consequences are emerging among vulnerable groups of population. Migrants and refugees face particular challenges in regards to the current COVID-19 crisis such as those related to access to information and to medical attention. Furthermore, as observed previously, undocumented migrants are less likely to seek care or tend to delay health care seeking due to their status (7, 8). This can be explained by the acute contradiction between considering one as sick and the necessity to keep working in the absence of any sick leave mechanism (12).

Undocumented migrants live in their country of destination as workers employed in informal and precarious job sectors (13), incompatible with teleworking promoted by the lockdown measures and in positions typically hindering the capacity to observe social distancing (2). Often active in domestic and care work, looking after the elderly and children at home, they provide a crucial contribution in the destination countries while depending on volatile daily wages (3, 8). Due to the necessity to provide for themselves, their family living with them and possibly their relatives in their country of origin through remittances (7), the absence of work equating with the absence of income has devastating consequences for different circles of people.

Their access to stable accommodation and sufficient supply of quality food has been shown to be jeopardized by increased precariousness in time of economic crisis (14). In the COVID-19 context, their poor housing conditions further affect their elevated risks of infection since common overcrowding limits the capacity to isolate oneself when infected (14, 15). The absence of income brings the additional fear of homelessness (3). The lockdown measures also impacted access to food. For instance, estimates pointed to a four-fold increase in the number of food insecure people in the UK since the beginning of the crisis (16). In Geneva, migrants have been severely affected forcing the authorities and community organization to set up large-scale emergency food distributions (17).

Overcoming multiple challenges, undocumented migrants have managed to stay in destination countries thanks to their capacity to conduct their life without any encounter with governmental institutions. Over time, they have learned to cope with recurrent difficulties through their own means and thus prefer avoiding any formal help for fear it would jeopardize their chance to obtain a legal status (8). Indeed they tend to avoid seeking assistance outside their immediate social circle by fear of denunciation and expulsion (18). In the COVID-19 context, official messages encouraging them to seek help have thus been received with suspicion, due to a pre-existing limited trust in the government. Furthermore, along their traditional exclusion from social safety mechanisms, they were not included in exceptional measures adopted to respond to the COVID-19 pandemic in the US (8). In Switzerland also, domestic work was excluded from the federal measures offered to the working population.

While undocumented migrants encompass individuals with distinct levels of resources and capacities, existing research shows that they tend to cumulate difficulties in their daily living and working conditions and to have limited access to different types of securities and resources that come along a residence status (19). Regularization of the residence status could theoretically entail better protection against social and health consequences of recessive economic cycles by fostering more resilience and resources. Set in the context of the COVID-19 crisis, this study aims at assessing whether undocumented migrants and migrants who have obtained a residency permit have been affected and have coped differently with the many challenges of the crisis. Better knowledge of such differences may allow for designing targeted health and social policies.

## Methods

### Setting

Undocumented migrants account for 10,000–15,000 of the 500,000 residents of the Canton of Geneva, Switzerland (20). In 2017, the local Council has implemented a conditional regularization policy (Operation Papyrus) that aimed to provide residency permit to undocumented migrants meeting the following criteria: (a) residence in Geneva for 10 years or 5 years for parents of children admitted at school; (b) financial independence; (c) social integration; and (d) lack of criminal record (21).

Switzerland was among the countries most rapidly and severely affected relative to its size by the COVID-19 epidemic in Europe during the March-April 2020 period (22). The first case was confirmed on February 26. Geneva along with Vaud and Ticino were the cantons most severely hit. The peak of incidence of new positive tests in Geneva was reached on March 30, 2020 with 280 cases. On April 20, 2020 when we launched our questionnaire, the incidence and mortality rates per 100,000 residents were 979.8 and 43.6 cases, respectively. In response, the Federal Government implemented swift measures from February 28, 2020 leading to a generalized lockdown by March 15, 2020. Control measures were progressively relaxed from April 27, 2020 with the reopening of few businesses, and on May 11, 2020 when primary schools re-opened and non-essential activities progressively re-started.

### Design

This mixed-methods study is nested within the prospective Parchemins Study which started in 2018 and aims at monitoring the impact of the Papyrus regularization policy on undocumented migrants' health and well-being (9). In short, the Parchemins Study collects qualitative and quantitative data annually among a cohort of migrants, whose initial sample included 464 participants recruited in the community and sharing similar background socioeconomic features but distinguished by legal status. The regularized group (*n* = 213) has successfully obtained a residency permit or is in the process of obtaining it while the undocumented group (*n* = 251) either lacks one or more regularization criteria or is unwilling to engage in such process. Details on inclusion criteria and recruitment into the Study can be found elsewhere (9).

### Participants

We invited all the Parchemins Study participants who had responded to the first two data collection waves (*n* = 379) to fill up an online questionnaire available in French, English, Spanish and Portuguese on April 20 (8 weeks after the first confirmed COVID-19 case in Switzerland). We closed the survey on May 10, 2020, the day before the second round of lockdown relaxation happened (Table 1). Invitation was sent by email and by short message texting. In absence of response, a second invitation was sent. The questionnaire was easily accessible on any electronic device connected to internet and was designed to take no more than 15 minutes to be filled. It was pre-tested with 5 persons before its implementation.

**Table 1:** Timeframe of the data collection in relation to the COVID-19 lockdown in Switzerland (March to June 2020).

**Period**	**February 28–March 15**	**March 16–April 26**	**April 27–May 10**	**May 11–June 7**	**June 8 onwards**
Mass gathering limitation	<1,000 persons	<5 persons	<5 persons	<5 persons	<300 persons
Circulation, economic activity		Borders, schools, and non-essential businesses or services closure	Partial reopening of few critical businesses and services	Primary schools and partial non-essential businesses reopening	Secondary schools, university, and businesses full reopening
Key message from authorities		Stay at home	Stay at home	Careful return to activity	Full return to activity
Online questionaire		April 19–May 10			
Telephone interviews		May 4–8			

### Questionnaire Variables

We explored sociodemographic, health, living conditions, economic, and employment-related variables. Self-rated health was measured by using the first of the 12-Item Short-Form Survey (SF-12) and we regrouped the answers “excellent” and “very good” as indicating the absence of reserve regarding one's health evaluation (23). We assessed the direct health impact of COVID-19 by aggregating those with a positive diagnostic test (confirmed cases), those with evocative symptoms and close contacts with a confirmed case and those asymptomatic who had been asked by health authorities to self-isolate after a contact with a confirmed case. Satisfaction with life was included as an indicator of well-being. Participants had to grade their current level of satisfaction on a 10-point Likert scale. We also assessed participants' main immediate concerns in the pandemic context. They could provide several answers which were regrouped into five main categories (health, employment and income, situation in their country of origin, living conditions in Geneva and social isolation).

We assessed different types of difficulties related to basic needs. We screened for financial insecurity by asking about the ability to pay a CHF 1,500 (Euro 1,450, $ 1,500) bill at short notice. This question is used in population surveys in Switzerland with a higher threshold (CHF 2,500), but adjusted in our survey as the median income of undocumented migrants is well below the one of the general Swiss population (24). We are aware this level fares much higher than in most contexts, as for example in the United States, 40% of adults could not pay for a $400 emergency expense (25). Food insecurity was assessed by asking participants the following questions: “since the beginning of the COVID-19 crisis, have you had to purchase cheaper or lesser quality food?” and “since the beginning of the COVID-19 crisis have you had to eat less or to skip meals because you were running out of food?.” Food insecurity was considered present in case of a positive response to one of the two items. We then graded the severity of food insecurity by distinguishing those suffering from hunger from those who had not suffered from hunger. These items were adapted from the Short Form of the Household Food Security Scale (26). Housing insecurity was defined by the fear of losing the current accommodation at short notice and financial insecurity by the reduction or the absence of income.

### Statistical Analysis

Non-normally distributed continuous variables are presented as median and interquartile range (IQR), whereas categorical variables are listed as absolute number proportions and percentages. Continuous variables were compared with the Mann-Whitney test. Categorical variables were compared with the chi-square test or the Fisher's exact test, as appropriate. Considering that this study was purely descriptive and did not intend to determine causal relationships between the factors under observation, we presented the results of all analysis including those including missing data. The significance level was set at 0.05. All analysis were conducted using IBM SPSS Statistics version 26.

### Qualitative Interviews

With the intention to obtain more in-depth information on the coping mechanisms put in place, participants were asked if they would be available for a short telephone interview. Among those who accepted, we selected a purposive subsample, looking for diversity in terms of gender, origin and status (undocumented vs. regularized), while focused on those who had reported financial or food insecurity in the questionnaire. The interview guide was designed on the basis of the preliminary results of the quantitative data. A specific emphasis was put on how they allocated their available resources and their likelihood of seeking (or not) external support from their friends and relatives, community, employers and the non-governmental associations or official institutions. Interviews were conducted May 4–8, 2020 once most of the quantitative data collection was done. They lasted between 20 and 75 minutes; they were conducted in French, English, Spanish and Portuguese.

These interviews were recorded and fully transcribed. A deductive form of analysis was driven by key themes assessed in the quantitative survey and transcripts were coded accordingly in terms of encountered difficulties (main concerns, financial, and food insecurity), available resources and assistance-seeking. Inductive analysis was also conducted to explore coping mechanisms, social services uptake, and barriers to services uptake and transcripts were thus coded according to emerging themes. In the result section, interview citations will be used to extend and illustrate quantitative findings.

### Ethics Approval and Consent to Participate

All participants provided inform consent and this study was approved by the Ethics Committee of Geneva Canton, Switzerland (CCER 2017–00897).

## Results

### Participants

A total of 117 (30.9%) of the 379 Parchemins study participants responded to the questionnaire. Two persons had responded after the closure of the survey and were excluded as the lockdown had already been partially lifted, which may have impacted the answers. Five participants could not be appropriately identified as regard their legal status and 2 had not fully completed the questionnaire. We thus included 108 (92.3% of the 117 respondents and 23.3% of the whole study population) participants in the analyses. Compared to participants who had responded in the second study wave (2019) but not to the COVID-19 survey, the 2020 sample was similar in terms of gender distribution, age, origin, and legal status. Respondents and non-respondents to the COVID-19 survey were also comparable in terms of health profile at the time of the second data collection (Appendix 1).

More than two-thirds of the participants were regularized or in the process of regularization and a majority of them were middle-aged women from Latin-America (Table 2). Undocumented migrants were younger than their regularized counterparts. Only a minority of all participants had children at charge in Geneva.

**Table 2:** Demographics, health and living conditions of participants (*n* = 108).

	**Total (*N* = 108) *n* (%) or median (IQR)**	**Missing value *n* (%)**	**Undocumented (*N* = 31) *n* (%) or median (IQR)**	**Regularized(*N* = 77) *n* (%) or median (IQR)**	*p*-value^*^
Women	85 (78.7%)	0 (0%)	23 (74.2%)	58 (80.5%)	0.468
Origin		0 (0%)			0.060
Latin America	68 (63.0%)		14 (45.2%)	54 (70.1%)	
Asia	27 (25.0%)		12 (38.7%)	15 (19.5%)	
Non-EU/EFTA Europe	5 (4.6%)		1 (3.2%)	4 (5.2%)	
Africa	8 (6.1%)		4 (12.9%)	4 (5.2%)	
Age (years)	47.7 (16.3)	0 (0%)	41.8 (15.8)	49.2 (14.8)	0.003
Children at charge in Geneva	16 (14.8%)	0 (0%)	4 (12.9%)	12 (15.6%)	0.723
Health impact of COVID-19	13 (12.4%)	3 (2.8%)	5 (16.7%)	8 (10.5%)	0.512
Self-rated health (very good or excellent)	51 (49.0%)	4 (3.7%)	14 (48.3%)	37 (49.3%)	0.923
Self-rated health deterioration since COVID-19 lockdown	14 (13.9%)	7 (6.5%)	6 (20.7%)	8 (11.1%)	0.218
Feeling of anxiety or depression	70 (68.0%)	5 (4.6%)	20 (71.4%)	50 (66.7%)	0.645
Avoidance of health care since the beginning of the lockdown	37 (35.9%)	5 (4.6%)	15 (53.6%)	22 (29.3%)	0.023
Employed before the lockdown	93 (86.9%)	1 (0.9%)	25 (80.6%)	68 (89.5%)	0.224
Reduction in working hours among those still employed since the lockdown	69 (74.2%)	0 (0%)	19 (76.0%)	50 (73.5%)	0.809
Sending remittances to country of origin	54 (51.4%)	3 (2.8%)	12 (41.4%)	42 (55.3%)	0.203
Ability to cover a 1,500 CHF unexpected expense	21 (20.2%)	4 (3.7%)	3 (10.3%)	18 (24.0%)	0.120
Financial resources for ≥3 months	23 (22.3%)	5 (4.6%)	6 (20.0%)	17 (22.3%)	0.716
Satisfaction with life (0–10)	7 (4)	5 (4.6%)	6 (4)	7 (4)	0.068

* *Refers to the comparison between undocumented and regularized groups*.

Seventeen of these participants were further interviewed by telephone, this subsample included 11 women and 6 men (median age 45.1). Among them, 9 had applied for regularization and 8 were undocumented.

### Health and Access to Care

Around one in eight participants had suffered direct health consequences of the COVID-19 infection: four had been tested COVID-19 positive and nine thought they had contracted it but had not been tested. Two had to be hospitalized and 13 self-isolated at home during at least 10 days. A large majority declared feeling anxious or depressed in relation to the current global situation and around one in eight thought their health had worsened since the beginning of the COVID-19 crisis. Half of the respondents rated their health as very good or excellent. A majority of undocumented participants had avoided health care since the beginning of the COVID-19 crisis, which was significantly more than in the regularized group (Table 2).

### Living Conditions and Satisfaction With Life

In total, 86.9% participants were employed before the crisis' onset and three in four employees had witnessed a partial or total loss of working hours in the last 2 months. Only 22.3% declared having enough financial resources to cover their basic needs for the next 3 or more months if the lockdown was to continue and a similar proportion was able to pay an unexpected CHF 1,500 bill at short notice. Yet, more than half kept sending remittances to relatives in their country of origin. There was no statistically significant differences between the two groups. Overall, undocumented migrants tended to rate their satisfaction with life lower than those regularized.

### Main Preoccupations During the COVID-19 Crisis

**Figure 1:**
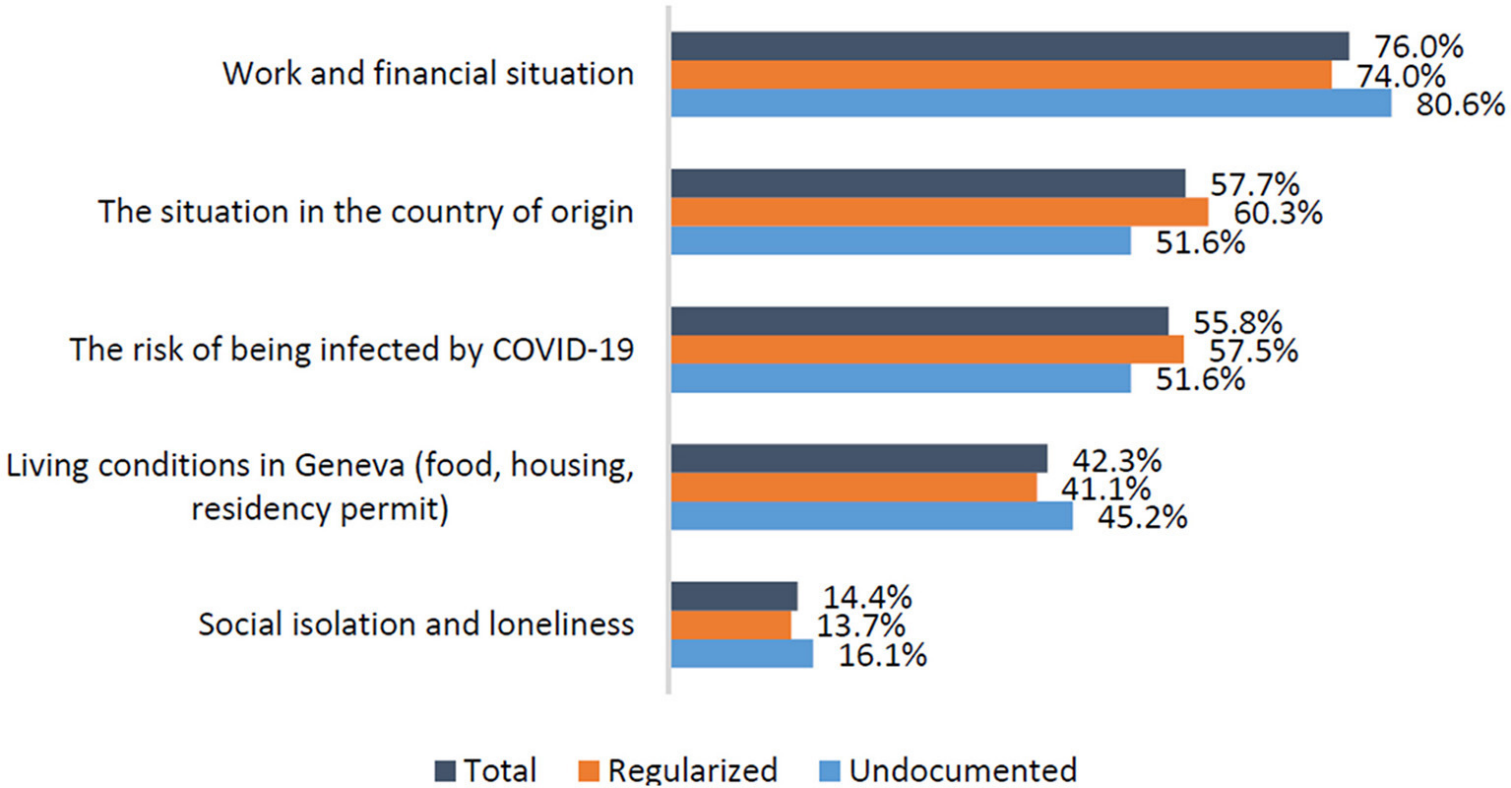
Main current preoccupations (several answers possible) (*n* = 108, missing values = 4).

A large majority of participants rated the loss of employment and income as their main immediate concern during the lockdown (Figure 1). The political and health situation in the country of origin and the risk of COVID-19 infection in Geneva were also important preoccupations but at a lower level. Living conditions and social isolation were less frequently mentioned. These preoccupations were reported in similar proportions by the 2 groups. The immediate economic impact of the lockdown has been detailed in the telephone interviews, as illustrated by this participant:

*My main concern is that I lost my job. I have just been fired… I have only 20% of my budget left… They [employers] don't want me to come to their house because of COVID. She said she is now staying at home and can take care of the children herself… Without any advance notice… I don't earn any income anymore, and receive no assistance… I only live on my savings. I am not eligible for unemployment benefits… It's very hard* (Brazilian woman, 28 years old, waiting for residency permit).

### Presence and Accumulation of Difficulties

Financial, housing, and food difficulties were all highly prevalent (Table 3). More than two thirds of participants presented financial or housing insecurity and 61% declared being food insecure. Among those suffering food insecurity, one quarter had been hungry but decided not to eat by lack of financial resources since the beginning of the lockdown. Undocumented migrants were significantly more exposed to housing and food insecurity than regularized migrants. The tension between paying one's rent and still having money to buy food has been raised in several interviews:

*This [COVID-19 context] affects me a lot. This is the reason I was thinking of asking for some food assistance. Because while I am paying my rent, I cannot also pay for food* (Bolivian woman, 45 years old, undocumented).

*Food is not my main concern. My problem is that I have just been able to pay my rent at the end of April. However, I will not be able to do so for the month of May. It is more critical for me to pay the rent than to care for food* (Brazilian woman, 50 years old, residency permit).

**Table 3:** Prevalence of difficulties among migrants (*n* = 108).

	**Total (*N* = 108) *n* (%)**	**Missing values *n* (%)**	**Undocumented (*N* = 31) *n* (%)**	**Regularized (*N* = 77) *n* (%)**	*p*-value^*^
Financial insecurity	76 (70.4%)	0 (0%)	24 (77.4%)	52 (67.5%)	0.309
Housing insecurity	72 (69.9%)	5 (4.6%)	23 (85.2%)	49 (64.5%)	0.044
Food insecurity	64 (61.0%)	3 (2.8%)	24 (77.4%)	40 (54.1%)	0.025
Food insecurity with hunger	15 (23.4%)	0 (0%)	7 (29.2%)	8 (20.0%)	0.402

* *Refers to the comparison between undocumented and regularized groups*.

**Figure 2:**
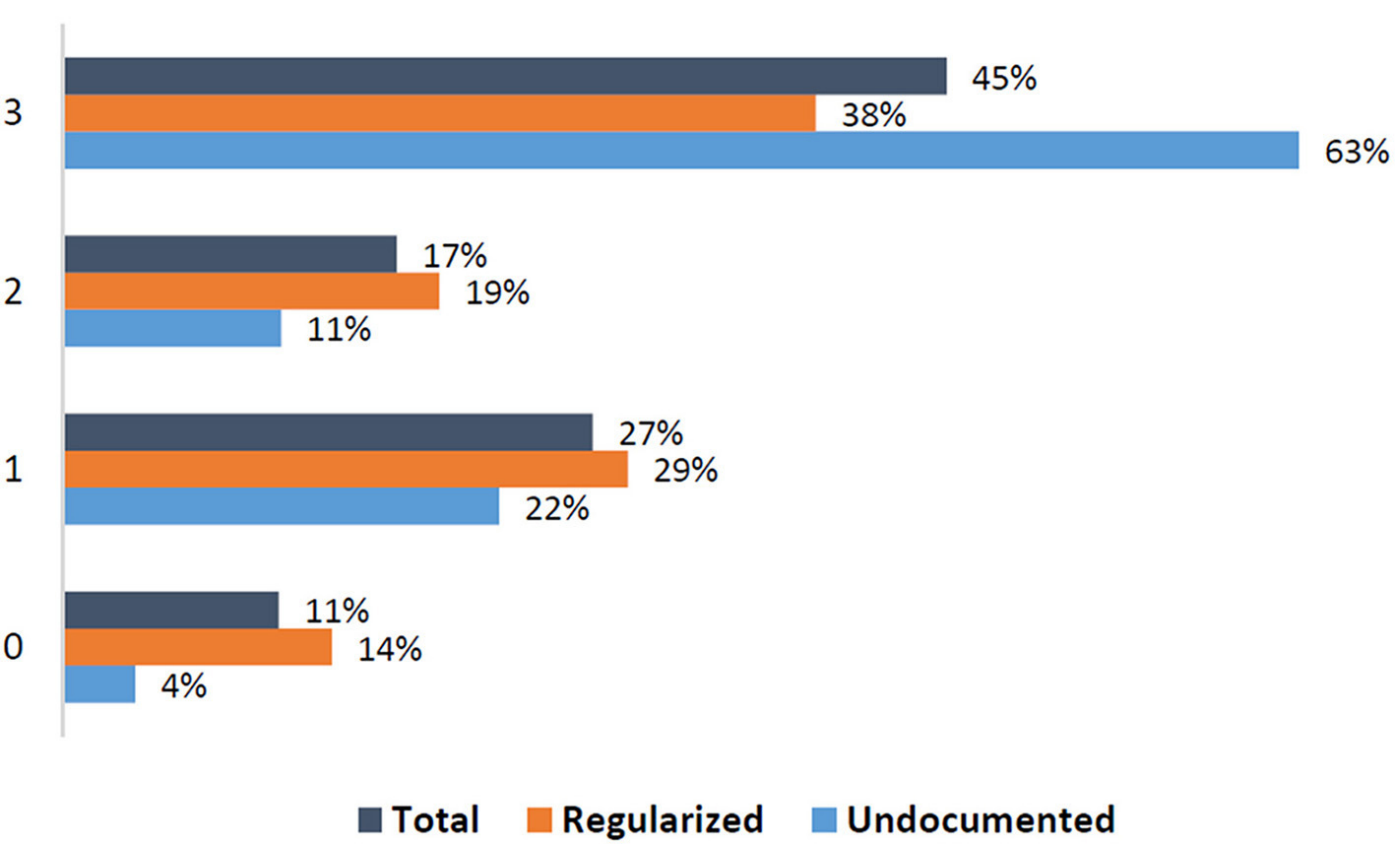
Number of difficulties among migrants (*n* = 108, missing values = 8).

Overall, participants reported a median of 2 (IQR = 2) difficulties with significant difference between undocumented (3; IQR = 2) and regularized (2; IQR = 2) migrants (*p*-value 0.032) (Figure 2).

### Assistance-Seeking Behavior

A total of 51.9% of participants had engaged in seeking external support to fulfill their financial, material and food needs (Table 4). Undocumented migrants reported doing it more frequently, while not significantly. The main strategy was to request a financial loan from the immediate social circle of friends or relatives, while asking for support from one's employers or a public or non-governmental organization was less frequent. Regularized migrants tended to specifically avoid the latter source of support. Solidarity among undocumented migrants was expressed in this interview: “*My friend helps me a lot. He often offers me to join him for diner and as soon as he receives his salary, he gives me 50 or 100 francs [CHF].”* (Senegalese man, 42 years old, undocumented). While many employers quit paying wages during the lockdown, some continued to do so and allowed to cope with the situation: “*Most of my employers continue to pay me even though I am not working for them. Only two have stopped. This helps me a lot.”* (Bolivian woman, 45 years old, undocumented).

**Table 4:** Types of assistance and reasons for not seeking assistance (*n* = 108).

	**Total (*N* = 108) *n* (%)**	**Missing values *n*(%)**	**Undocumented (*N* = 31) *n* (%)**	**Regularized(*N* = 77) *n* (%)**	*p*-value^*^
Have sought assistance	54 (51.9%)	4 (3.7%)	19 (63.3%)	35 (47.3%)	0.138
Loan from friends or relatives	31 (29.5%)	3 (2.8%)	13 (43.3%)	18 (24.0%)	0.050
Financial, material or food support from employer	21 (20.2%)	4 (3.7%)	7 (22.6%)	14 (19.2%)	0.693
Financial, material or food support from governmental or non-governmental organization	18 (17.3%)	4 (3.7%)	8 (27.6%)	10 (13.3%)	0.085
Main reason for not seeking assistance (*n* = 93)		6 (6.5%)			0.003
Considered as non-necessary	38 (43.7%)		4 (17.4%)	34 (53.1%)	
Fear of not obtaining or renewing a residency permit	27 (31.0%)		8 (34.8%)	19 (29.7%)	
Prefer managing by oneself	22 (25.3%)		11 (47.8%)	11 (17.2%)	

* *Refers to comparison between undocumented and regularized groups*.

Half of those who had not sought help said it was not necessary, one third were worried about the adverse consequences this could have on their legal status and one quarter reported they preferred to manage by themselves (Table 4). This was expressed by this participant: “*I want to find more working hours, at least one job, so that I do not ask help from anyone. I prefer to work*” (Bolivian woman, 66 years old, waiting for residency permit). Reasons for not seeking assistance significantly differed between the two groups: while undocumented migrants reported preferring to manage by themselves, regularized migrants mainly mentioned that it was not necessary. Both groups feared the potential administrative consequences of applying for public support on their ability to receive or renew their residency permit in the near future in similar proportions. This fear was reported on several occasions during the interviews:

*I haven't sought any assistance because this would hinder the renewal of my residency permit. When we apply for a permit, we always hear* ≪*be careful, don't ask for any social assistance because if they learn about, they will deny the permit renewal*≫ (Brazilian woman, 51 years old, residency permit).

*I did not seek assistance because I don't want that it stays in my record* (Brazilian woman, 40 years old, undocumented).

Quantitative data mostly collected 4–5 weeks after the initiation of the lockdown measures highlight the capacity and willingness of this population to handle the economic and material impact of the COVID-19 crisis on their own, or through informal sources of help. Compared to the survey, the qualitative interviews, conducted over 6 weeks after the beginning of the lockdown showed how financial hardship increased with time and how migrants were facing further difficulties as an additional rent had to be paid.

*Last month, it was ok. I did not have any problem, but this month of May, it is more complicated… I have to limit myself in many things, to be able to make it. But I feel it is worse* (Bolivian woman, 45 years old, undocumented).

Interviews also showed how coping strategies changed over time. Solidarity among vulnerable migrants declined as a result of the lasting situation: “*None of my friends can lend me money as they are all facing difficulties. They do not have any money left either*” (Indian man, 35 years old, undocumented). This led some to consider looking for help from governmental or non-governmental sources:

*It is tight now. I eat and pay what I am able to for the time being. If the situation stays unchanged, I will have to seek assistance, be it to the Colis du Coeur [food distribution] or at the Hospice général [social aid institution]. I know I will need help* (Brazilian woman, 50 years old, residency permit).

While participants acknowledged during the interviews that they considered looking for help, they also reported a range of barriers hampering uptake. Access problems were discussed: information was not easily available and well-known social services were closed due to the lockdown: “*I did not know where to go and nobody could tell me, above all with regard to food assistance*” (Bolivian woman, 66 years old, waiting for residency permit). Language barriers and procedures also hindered possibilities to be helped:

*I just tried to call the Hospice général but this was difficult. They answer in French and I don't understand. I sent an email but they cannot answer because they say this is confidential*. (Filipina woman, 61 years old, residency permit).

In addition, the complexity of assistance mechanisms was highlighted. While she was aware of her employer's obligation to pay her wages despite the lockdown, this participant prioritized finding a job over claiming her rights:

*I know I could ask for the recognition of my rights [to be paid by the employer]. However, this takes time. I have my rent to pay, my food, my phone… It is always the same story: either you fight for your rights, or you eat !… I need to work this week, that's it* (Brazilian woman, 28 years old, waiting for residency permit).

Ineligibility or illegitimacy were an additional source of renouncement to assistance: *We don't feel legitimate to ask for assistance because we are illegal here. I try to stay invisible and I don't know whether there is any assistance for people like me* (Algerian man, 39 years old, undocumented). Finally, feelings of shame or indignity were also reported as reasons to not ask for any help:

*The Colis du cœur [food distribution]? Yes, I know about it. But no, no… For me it is not honorable… no. Sorry… it is undignified* (Indian man, 35 years old, undocumented).

*For us, it is shameful… My colleagues from work have kids, and the situation is really hard for them. But still, they will not ask for assistance…. I tell them it is better than to starve, but still they say no* (Kosovar man, 36 years old, residency permit).

Interviews thus emphasized the tension between the important consequences of the lockdown and the multi-fold barriers to support, including ideas about not deserving to receive any formal assistance.

## Discussion

This study provides empirical evidence on the early and severe health and socioeconomic impact of the COVID-19 pandemic and the associated lockdown in spring 2020 on undocumented or recently regularized migrants in a high-income country. Over the mixed data collection period, the accumulation and rapidly progressing difficulties in their living conditions could be observed. The duration of the public health measures put them at risk of not being able to meet their basic needs, as access to work and its associated income was limited.

This is the first study in Europe to directly document the social and health impact of the COVID-19 crisis among this hard-to-reach group. The more frequent exposure to COVID-19 than in the general population, associated with housing difficulties, confirms the specific risks this group faces in the ability to adopt preventive and protective measure (2, 8). Indeed, the capacity to respect social distancing is socially distributed along teleworking opportunities (27). Most participants in our study work in jobs (domestic or construction work) requiring their presence, which added to their dense housing conditions, puts them at higher risk of infection than the general population, across a number of settings. Their frequently limited access to medical care may further fuel this dynamic of transmission (8, 27). In the COVID-19 context, access to the public hospital has been tightly channeled by security staff, with the likely consequence to repel undocumented migrants.

In presence of different sources of preoccupations, including acquiring COVID-19, migrants frequently presented poor psychological health. It can be hypothesized that besides concerns about their own health, the fear of not being able to generate a sufficient income to satisfy their own and their family' basic needs in Geneva and to continue supporting their family back home in a period of deep global economy downturn may be sources of acute and severe stress. Yet, even though a majority of participants had experienced a sharp reduction in their income, more than half were still sending remittances back home which may be seen either as a source of supplementary stress or as a way to show resilience by maintaining one's role of breadwinner. Of interest, while nearly half of the participants declared some reserve regarding their actual health, only a minority mentioned it had worsened since the beginning of the pandemic. This may reflect the focus on the somatic dimension of health when precarious migrants are asked to self-rate their health. Our findings highlight the need for universal health coverage for undocumented migrants with an emphasis on mental health services taking into account the specific circumstance they are facing in order to reduce the further health consequences and strengthen their capacity of resilience (28–30). This is particularly important considering results showing that in the COVID-19 context poor mental health is related with the avoidance of health care (31).

We found migrants faced cumulative and evolving difficulties to meet their basic needs in the context of financial hardship. Food insecurity often overlapped with other types of difficulties. It can be postulated that food insecurity may represent the tip of the insecurity iceberg in this group as, unlike the rent or the medical insurance, it is not a fixed and regular expense. Our data suggests migrants may adopt trade-off strategies to preserve what they consider as essential securities such as housing by cutting on other less urgent expenses such as medical care and food. Indeed, a very tight local housing market like in Geneva makes homelessness a concrete preoccupation. Of concern, a proportion of participants had already severely cut on food expenses and declared themselves as hungry which may both affect their health and well-being and their ability to rapidly bounce back once the lockdown is lifted.

While the two groups of participants had similar exposure to financial insecurity, undocumented migrants reported significantly more often housing and food insecurity. This is not explained by differences in assistance seeking behaviors as regularized migrants tended to renounce to sources of support more frequently. Rather, it may suggest the latter had more reserves to face a sudden drop in income and could better tackle the situation of crisis thanks to the resources accumulated previously. Yet, only 22.3% declared having enough resource to meet their basic needs for 3 or more months, which indicated that even though regularization may entail better protection against short-term difficulties, mid-term perspectives remained bleak. The fact that financial autonomy is a demand for any permit application or renewal constitutes a major barrier to assistance uptake for migrant workers in time of crisis.

Overall, this population had clearly internalized that looking for formal assistance could jeopardize their chances to become regularized. This reluctance to seek support reflects their long-standing capacity to manage on their own. Indeed over time, undocumented migrants develop skills for camouflage in order to live, work, send their children to school in the hope to eventually get access to a legal status (32). In this study, the qualitative interviews notably offered additional insights on the rationale behind the norm of autonomy emphasized by the quantitative results: these workers have managed to stay in the destination country thanks to their ability to not interact with formal structures. Interviews suggested that the length of the lockdown measures was however putting more and more pressure on limited available resources, thus making assistance-seeking inevitable despite its perceived risks and downsides.

This study has some limitations to account for. First, it only reflects the situation at a specific time point during the COVID-19 lockdown within a period marked by rapid epidemiological, economic and political evolutions. The time lapse between the survey and the qualitative interviews provides an insightful illustration of potential variations both in terms of evolving difficulties and coping mechanisms over a relatively short period. Second, data was collected with a limited sub-sample recruited from our larger cohort study; however considering the general difficulty to reach this population, we consider they offer valuable information in a context making empirical research particularly challenging. In addition, the different environments in neighboring countries prevent from generalizing our findings to all undocumented or recently regularized migrants in Europe. Yet, we believe our findings honestly describe the situation in Switzerland in the midst of the COVID-19 lockdown given the bond of trust built with the study participants along the first two waves of data collection.

As in our larger longitudinal project, we consider quantitative and qualitative data usefully complement each other to understand the trajectories and preoccupations of undocumented migrants. In the present study, interviews helped to better grasp their stressful conditions, especially since these were rapidly changing as the lockdown consequences accumulated over weeks. The strength of mixed methods lies in its combined capacity to offer quantitative findings providing support to public policies elaboration and qualitative material giving insights on how people's consider their own circumstances, allowing to develop interventions aligned with their actual needs.

## Conclusion

The cumulated difficulties faced by migrants in this period of crisis and their limited search for assistance highlight the need to implement trust-building strategies to bridge the access gap to governmental and non-governmental sources of support. Indeed, migrants in irregular situation have lived for years with the necessity to hide from authorities and have internalized that calling for support may be detrimental to the provision or the renewal of a residency permit. In Switzerland, even though the Federal Government publicly declared that seeking assistance would not penalize migrants in their future demand for regularization, very few of them felt confident to seek support, confirming the statement that it is naive to think this population will trust authorities in such unprecedented time (2).

These findings emphasize the importance of policy measures addressing both human rights and public health considerations. Policies that may prevent or mitigate the burden of poor health and life difficulties among migrants during COVID-19 like pandemics in Western countries may include: (a) enforcing the legal framework about the working conditions in order to protect employees of informal sectors from unfair dismissal and sudden loss of income without compensation; (b) ensuring that migrants with different legal situations access effective and rapidly accessible sources of financial, material, and food support during the economic crisis without risk for their current or future application for a residency permit; (c) facilitating access to temporary residency permit and to social security as long as the economic crisis puts undocumented migrants at consequent risks of not being able to meet their basic needs; and (d) continuing to monitor the situation as the context evolves rapidly and subsequent waves of widespread dissemination of the infection within the population may again cause severe economic consequences. By contributing to the overall public health response of mitigating the spread of the COVID-19 virus, these measures can also avoid vulnerable groups, like undocumented or recently regularized migrants, to be blamed and discriminated.

The long lines of people waiting for food distribution made these groups highly visible, locally and internationally, against their will. This publicity offers a chance to emphasize the structural conditions that allow undocumented migrants to stay, i.e., needs in the economic market for cheap and unprotected labor while their presence is officially denied. The COVID-19 pandemic and the associated measures to slow the progression of the virus have somehow acted like a real life test of the importance to be regularized, as opposed to have no residency permit. Our findings indicate that regularization matter: the local regularization policy put in place in Geneva helped those who had obtained a legal status to better cope with the consequences of the COVID-19 crisis. However, even if they were less affected, their position remains fragile.

## Data Availability Statement

The raw data supporting the conclusions of this article will be made available by the authors, without undue reservation.

## Ethics Statement

The study was reviewed and approved by Geneva Canton board of ethics. The participants provided their written informed consent to participate in this study.

## Author Contributions

CB-J: conceptualization, methodology, and original draft. AD: investigation, data curation, and analysis. SL: investigation, analysis, and original draft. LC and JF: methodology, review, and editing. YJ: conceptualization, supervision, and original draft. All authors contributed to the article and approved the submitted version.

## Conflict of Interest

The authors declare that the research was conducted in the absence of any commercial or financial relationships that could be construed as a potential conflict of interest.
