# Supplementary material for: The Impact of the Covid-19 Pandemic and the Lockdown on the Health and Living Conditions of Undocumented Migrants and Migrants Undergoing Legal Status Regularization
Source: Front Public Health. 2020 Dec 16;8:596887. doi: 10.3389/fpubh.2020.596887 (PMC7772178; doi:10.3389/fpubh.2020.596887)
Supplement: Supplementary file 1 [file Table_1.pdf]

Appendix 1: Demographic and health characteristics of respondents and non-respondents to the survey

	Respondents to the Parchemins Covid Survey (N=108)	Non-respondents to the Parchemins Covid Survey (N=271)	
	n (%) or median (IQR)	n (%) or median (IQR)	p-value
Women	85 (78.7%)	193 (71.0%)	0.124
Age (years)	45.2 (14.6)	47.7 (16.3)	0.161
Origin			0.345
Latin America	68 (63.0%)	183 (67.3%)	
Asia	27 (25.0%)	53 (19.5%)	
Non EU/EFTA Europe	5 (4.6%)	22 (8.1%)	
Africa	8 (6.1%)	14 (5.1%)	
Undocumented	31 (28.7%)	66 (24.3%)	0.371
Self-rated health (very good or excellent) in wave 2	48 (44.4%)	96 (35.3%)	0.097
Feeling of anxiety or depression in wave 2	28 (25.9%)	53 (19.5%)	0.167
